# A Bacterium Isolated From Soil in a Karst Rocky Desertification Region Has Efficient Phosphate-Solubilizing and Plant Growth-Promoting Ability

**DOI:** 10.3389/fmicb.2020.625450

**Published:** 2021-02-01

**Authors:** Jinge Xie, Zongqiang Yan, Guifen Wang, Wenzhi Xue, Cong Li, Xiwen Chen, Defu Chen

**Affiliations:** ^1^Department of Genetics and Cell Biology, College of Life Sciences, Nankai University, Tianjin, China; ^2^Department of Biochemistry and Molecular Biology, College of Life Sciences, Nankai University, Tianjin, China

**Keywords:** phosphate-solubilizing bacteria, plant growth-promoting, *Acinetobacter*, karst rocky desertification, organic acid

## Abstract

Phosphorus in the soil accessible to plants can easily be combined with calcium ion, the content of which is high in karst rocky desertification (KRD) regions, thereby resulting in a low utilization efficiency of phosphorus. The application of phosphate-solubilizing bacteria (PSB) from the KRD region would facilitate enhanced phosphate availability in the soil. In the present study, the strains belonging to *Acinetobacter*, *Paraburkholderia*, and *Pseudomonas* with efficient phosphate-solubilizing ability were isolated from fruit tree rhizosphere soils in KRD regions. Particularly, *Acinetobacter* sp. Ac-14 had a sustained and stable phosphate-solubilizing ability (439–448 mg/L, 48–120 h). Calcium carbonate decreased the phosphate-solubilizing ability in liquid medium; however, it did not affect the solubilization index in agar-solidified medium. When cocultivated with *Arabidopsis thaliana* seedling, Ac-14 increased the number of lateral roots, fresh weight, and chlorophyll content of the seedlings. Metabolomics analysis revealed that Ac-14 could produce 23 types of organic acids, majorly including gluconic acid and D-(-)-quinic acid. Expression of Ac-14 glucose dehydrogenase gene (*gcd*) conferred *Pseudomonas* sp. Ps-12 with a sustained and stable phosphate-solubilizing ability, suggesting that the production of gluconic acid is an important mechanism that confers phosphate solubilization in bacteria. Moreover, Ac-14 could also produce indole acetic acid and ammonia. Collectively, the isolated Ac-14 from KRD regions possess an efficient phosphate-solubilizing ability and plant growth-promoting effect which could be exploited for enhancing phosphorus availability in KRD regions. This study holds significance for the improvement of soil fertility and agricultural sustainable development in phosphorus-deficient KRD regions.

## Introduction

There are three karst centers worldwide: in the European Mediterranean, the Dinaric karst regions of the Balkan Peninsula, and in Southwest China. In karst regions, the processes of soil erosion, vegetation loss, exposure of rocks, and the appearance of a rocky landscape are termed as karst rocky desertification (KRD, Wang et al., 2004; Jiang et al., 2014). KRD is considered as one of the most severe ecological problems worldwide (Wang et al., 2004; Tang et al., 2019). The degraded environment and low productivity in KRD regions hinders local economic growth and affects the people living in these regions (Jiang et al., 2014). Karst topography is a special landscape that is developed on carbonate rocks such as limestone, dolomite, or marble, and rocky desertification mainly occurs in carbonate rocks areas. Bare carbonate rocks generally produce Ca^2+^ and HCO_3_^–^ in soil (White, 1997), wherein Ca^2+^ can combine with H_2_PO_4_^–^ to form insoluble phosphate, which is inaccessible for plants.

Phosphorus (P) is one of the major essential macronutrients for plants and is an indispensable component of nucleic acids (RNA and DNA), proteins, phospholipids, and cofactors (such as ATP). To provide plants with nutritional requirements, P generally relies on the application of chemical P fertilizers to the soil. Nevertheless, these chemical P fertilizers are easily fixed by Ca^2+^, Fe^3+^, and Al^3+^ in the soil, thereby resulting in low utilization efficiency (Wei et al., 2018); on the other hand, extensive usage of chemical P fertilizer may lead to numerous environmental issues, such as soil compaction and water pollution, and may increase economic burden (Sharma et al., 2013; Wang et al., 2019). Considering the issue arising due to low efficiency of chemical P fertilizers, it is crucial to improve P availability in soil, particularly in the KRD regions.

Total P is abundant, but available P is generally low in soil (Wei et al., 2016). We previously reported that the community structure of rhizospheric bacteria is remarkably influenced by water-soluble phosphorus (WSP) in rocky desertification areas (Xie et al., 2019). Microorganisms play a pivotal role in the cycle of soil nutrients, wherein the phosphate-solubilizing bacteria (PSB) can convert insoluble phosphates into soluble forms that are available for plants. Recently, low-input agriculture has gained immense interest from researchers, with focus given to the development and use of commercial biological inoculants to the increase the availability of key nutrients, particularly P, to crop plants (Owen et al., 2015). PSB have been isolated from various environmental areas, including solid waste compost, metal-contaminated soil, activated sludge, and saltern sediments (Park et al., 2011; Zhu et al., 2011; Wei et al., 2016; Yu et al., 2019); however, no report is available regarding PSB isolated from the fruit tree rhizosphere soil in KRD regions. As native PSB have the advantage of colonizing in the local soil (Wang et al., 2018), PSB strains isolated from KRD soil would, therefore, have special significance and could be utilized in KRD ecological restoration and agricultural development.

The mechanisms of PSB on phosphate solubilization are complex. The redox activity of microorganisms, production of CO_2_, secretion of siderophores, enzymes, and organic acid, and nitrogen assimilation were considered to be PSB mechanisms that could transform insoluble P to soluble forms (Rodríguez and Fraga, 1999; Sharma et al., 2013; Owen et al., 2015). In general, production of low molecular weight organic acids is the main phosphate-solubilizing mechanism of PSB (Rodríguez and Fraga, 1999; Ludueña et al., 2018). Gluconic acid has immense importance and is mainly produced by the glucose dehydrogenase (GCD) which is encoded by *gcd* gene (Liang et al., 2020). Besides the solubilizing insoluble phosphates, most PSB can also produce plant growth-regulating substances, such as indole acetic acid (IAA) and ammonia, to promote plant growth (Ludueña et al., 2018; Wang et al., 2019).

To develop and restore the agricultural and ecological environment in KRD regions, it is necessary to isolate PSB strains that are suitable for the native soil that also present an efficient phosphate-solubilizing ability and plant growth-promoting effect in KRD soil. The present study aimed to isolate novel PSB strains from the rhizosphere soil of fruit trees in the KRD regions in Southwest China and to analyze characteristics and mechanisms of phosphate solubilization and plant growth-promotion. In this study, we isolated a strain of PSB (*Acinetobacter* sp. Ac-14) from the fruit tree rhizosphere soils in the KRD region which could solubilize phosphate and promote plant growth and found that gluconic acid plays an important role in maintaining sustained and stable phosphate solubilization. Our findings will provide a theoretical foundation for understanding the phosphate solubilization mechanism of PSB and offer a basis for the application and development of ecofriendly, high-yield agriculture in KRD regions.

## Materials and Methods

### Collection of Soil Samples and Isolation of PSB Strains

Soil samples were collected from the rhizosphere soil of fruit tree in KRD and non-KRD (NKRD) regions (Table 1), according to the method described previously (Xie et al., 2019). After adding sterile distilled water (soil sample: water = 1:10, w/v), the samples were shaken at 180 r/min for 30 min under 28 ± 2°C. After serial dilution (10^–1^-10^–6^) with sterile distilled water, 100 μL of soil solution was placed on modified National Botanical Research Institute’s Phosphate (NBRIP) agar-solidified medium (Nautiyal, 1999, in Supplementary Material and Methods), and incubated at 28 ± 2°C for 6 days. The strains with clear halos were selected, halo zone diameter (D) and colony diameter (d) were determined, and solubilization index (SI) (the ratio of D/d) was calculated to roughly evaluate their phosphate-solubilizing capacities (Teng et al., 2019). The isolated strains were purified with modified NBPIP and AT salts medium (Nautiyal, 1999, in Supplementary Material and Methods) and stored with 20% glycerol in a refrigerator at −70°C.

**TABLE 1:** The soil samples and number of isolated and sequenced PSB strains from the fruit tree rhizosphere soil.

Soil No.	Region	Code	Latitude	Longitude	Altitude/m	County, province	Tree species	Tree age/year	No. of isolated PSB	No. of sequenced PSB	PSB species by 16S rDNA			
	
											*Ac.*	*Pa.*	*Ps.*	Others
1	KRD	EJ1	30°18′49″N	109°29′07″E	400	Enshi, Hubei	*Citrus reticulata*	3	39	2	0	0	2	0
2		GJ1	28°01′44″N	108°28′52″E	593	Yinjiang, Guizhou	*Citrus reticulata*	2	36	3	0	0	2	1
3		GJ2	28°01′46″N	108°28′55″E	588	Yinjiang, Guizhou	*Citrus reticulata*	2	43	11	**6**	0	0	5
4		GJ3	28°01′49″N	108°28′54″E	587	Yinjiang, Guizhou	*Citrus reticulata*	5	104	17	1	0	13	3
5		GY1	28°01′25″N	108°29′07″E	517	Yinjiang, Guizhou	*Citrus maxima* (Burm) Merr.	10	68	2	0	0	2	0
6		HT1	28°27′40″N	109°29′52″E	550	Huayuan, Hunan	*Amygdalus persica* L.	3	19	2	0	0	2	0
7		HZ1	28°26′33″N	109°28′49″E	550	Huayuan, Hunan	*Ziziphus jujuba* Mill.	3	17	4	0	0	4	0
8		NY1	28°12′38″N	112°35′42″E	388	Ningxiang, Hunan	*Citrus aurantium* L.	10	22	2	0	0	**2**	0
9		NY2	28°12′38″N	112°35′42″E	388	Ningxiang, Hunan	*Citrus aurantium* L.	10	17	1	0	0	0	1
10		WG1	30°46′16″N	114°12′24″E	21	Wuhan, Hubei	*Osmanthus fragrans* (Thunb.) Lour.	5	56	5	0	0	5	0
11		XB1	26°32′08″N	110°45′41″E	365	Xinning, Hubei	*Castanea mollissima* BL.	5	30	5	0	**4**	0	1
12		XQ1	26°39′46″N	110°57′52″E	427	Xinning, Huibei	*Citrus sinensis* L. Osbeck	10	59	2	0	0	1	1
13		XQ2	26°31′46″N	110°45′03″E	353	Xinning, Hubei	*Citrus sinensis* L. Osbeck	10	16	5	0	0	1	4
14		YH1	29°00′33″N	108°57′31″E	333	Youyang, Chongqing	*Zanthoxylum schinifolium* Sieb. et Zucc	5	27	0	0	0	0	0
15		YY1	28°56′38″N	109°56′38″E	330	Youyang, Chongqing	*Citrus maxima* (Burm.) Merr.	1	17	6	1	0	0	5
16	NKRD	CX1	43°54′13″N	125°20′31″E	187	Changchun, Jilin	*Armeniaca vulgaris* Lam.	10	59	11	0	0	11	0
17		CX2	43°54′13″N	125°20′37″E	211	Changchun, Jilin	*Armeniaca vulgaris* Lam.	20	107	8	0	0	8	0
18		CX3	43°49′13″N	125°16′41″E	251	Changchun, Jilin	*Armeniaca vulgaris* Lam.	10	69	3	0	0	2	1
Total		/	/	/	/	/	/	/	805	89	8	4	55	22

**KRD, karst rocky desertification; NKRD, non-karst rocky desertification; Ac, Acinetobacter; Pa, Paraburkholderia; Ps, Pseudomonas.**

### Identification and Characterization of the Isolated PSB Strains

16S rDNA sequencing was used to identify the isolated strains (Zhu et al., 2011; Biswas et al., 2018; Yu et al., 2019). Genomic DNA was extracted from the isolated strains and the 16S rDNA was amplified via polymerase chain reaction (PCR) using universal primers 27F (5′-agrgtttgatcmtggctcag-3′, where r, a or g; m, a or c) and 1522R (5′-aaggaggtgatccarccrca-3′). Each PCR reaction comprised 1.2 μL deoxynucleotide triphosphate (dNTPs, each 2.5 mM), 1 μL template DNA, 0.18 μL each primer (10 μM), and 0.075 μL Ex DNA polymerase (5 U/μL, TaKaRa Co., Ltd., Dalian, China). The products were detected via agarose gel electrophoresis and the 1.5 kb band was purified using a Gel/PCR extraction kit (Tiangen Biotech Co., Ltd., Beijing, China). The purified 16S rDNA fragment was ligated to pMD19-T Simple vector (TaKaRa Co., Ltd., Dalian, China), and then transformed into *Escherichia coli* DH5α competent cells. Positive clones were selected for sequencing, and the conservative sequences from numerous sequences were used for BLASTx with the NCBI database to identify the species. A phylogenetic tree was constructed using the neighbor-joining method with MEGA ver. X.

Strains were inoculated in Luria–Bertani (LB) medium (tryptone 10 g/L, yeast extract 5 g/L, and NaCl 5 g/L) and incubated overnight under 180 r/min at 28 ± 2°C. Thereafter, the cells were collected via centrifugation at 5,000 r/min for 5 min. After washing with phosphate buffer saline (NaCl 8 g/L, KCl 0.2 g/L, Na_2_HPO_4_ 1.44 g/L, KH_2_PO_4_ 0.24 g/L, pH = 7.4) 2–3 times, the samples were fixed with 2.5% glutaraldehyde at 4°C for at least 8 h, dehydrated gradually with 30, 50, 70, 80, 90, and 100% ethanol (twice), and then dissolved in absolute ethanol. The solution was dropped on the cover glass, dried at room temperature, and fixed to the scanning electron microscope (SEM) column after spraying gold. The morphology was scanned under SEM (QUANTA 200, FEI Company, Hillsboro, OR, United States). In total, 30 cells from each sample were randomly selected to measure the cell size using ImageJ. Gram staining was performed using the Gram stain kit (Beijing Solarbio Science and Technology Co., Ltd., Beijing, China).

### Evaluation of Phosphate-Solubilizing Characteristics of the Isolated PSB Strains

The sequenced PSB strains were incubated in LB medium until *A*_600_ = 1.8; thereafter, 10% (v/v) inoculum amount was transferred to liquid NBRIP medium and incubated under 180 r/min at 28 ± 2°C for 24 h. The cultures were centrifuged at 8,000 r/min for 10 min and the P content of the supernatant was detected via Mo-blue method (Murphy and Riley, 1962). The NBRIP medium inoculated with the same amount of LB was used as a negative control.

Moreover, the pH value of the supernatant and the *A*_600_ value of bacteria were determined at 6, 12, 24, 48, 72, 96, and 120 h after inoculation. pH was detected by a pH meter (PH400, Alalis Instruments Technology Co., Ltd., Shanghai, China). *A*_600_ value was measured by a spectrophotometer (TU-1810S, Beijing Purkinje General Instrument Co., Ltd., Beijing, China). To eliminate the effect of calcium phosphate in the culture medium on *A*_600_ value, the precipitate obtained by centrifuging the cultures was washed with equal volumes of 0.1 mM hydrochloric acid (Xiang et al., 2011).

Different concentrations of calcium carbonate (CaCO_3_) (0, 0.5, 1.0, 1.5, and 2.0 g/L) were added to NBRIP liquid medium to detect its effect on the growth of the isolated PSB strains. The SI value was evaluated on NBRIP agar-solidified medium containing CaCO_3_.

To evaluate the NH_4_^+^ assimilation of *Acinetobacter* sp. Ac-14, equal amounts of NH_4_Cl, NaNO_3_, and KNO_3_ were used to substitute (NH_4_)_2_SO_4_ in the NBRIP medium. After 24 h culture, the soluble P content, pH value, and *A*_600_ value of the supernatant of the cultures were determined.

### Evaluation of Plant Growth-Promoting Ability of the Isolated PSB Strains

*Arabidopsis thaliana* (Columbia) seeds were sterilized with 3% sodium hypochlorite for 1 min, washed with sterile water for 5–6 times, and sown on 1/2 Murashige and Skoog (MS) [Murashige and Skoog basal medium with vitamins (Duchefa Biochemie, NLD) 2.202 g/L, 2-(N-morpholino) ethanesulfonic acid (Genview, United States) 0.5 g/L and sucrose 10 g/L, pH = 5.7] agar-solidified medium. After preserving at 4°C for 3 days, the plates were vertically placed in a light incubator (22°C, 16 h light, 8 h dark) for 7 days. The seedlings were then transferred to NBRIP agar-solidified medium, and inoculated with PSB (10 μL, *A*_600_ = 0.05) around the roots. The plates were positioned vertically and cultured in a light incubator. After cocultivation for 7 and 14 days, the number of lateral roots, primary root length, fresh weight, and chlorophyll content of the seedlings were measured. The chlorophyll content was determined according to the method described by Xu et al. (2018). The seedlings inoculated with LB were used as the control.

### Untargeted Metabolomics of Ac-14

The Ac-14 strain was incubated in LB medium until *A*_600_ = 1.8, and thereafter, 10% (v/v) inoculum amount was transferred to liquid NBRIP medium and incubated under 180 r/min at 28 ± 2°C for 24 h. The cultures were then centrifuged at 8,000 r/min for 10 min, the supernatants were frozen in liquid nitrogen, and metabolomics were evaluated using liquid chromatography tandem mass spectrometry (LC-MS/MS) method by Novogene Bioinformatic Technology Co., Ltd. (Beijing, China). The detailed protocols are described in Supplementary Material and Methods. The supernatants from NBRIP medium inoculated with the same amount of LB were used as negative control.

### Cloning and Expression of the Ac-14 *gcd*

The genome of *Acinetobacter* sp. Ac-14 was sequenced by PacBio’s Single Molecule Real-Time (SMRT) sequencing technology. The sequence was submitted to NCBI GenBank (accession number CP063769). The *gcd* gene (2,406 bp, in Supplementary Material and Methods) was amplified via PCR using primers GCD-F (5′-agaattcatgaatcaaccta cttcaagatcagg-3′, underlined is *Eco*RI site) and GCD-R (5′-aggatccttatttgttatctggtaaggcataagcc-3′, underlined is *Bam*HI site). For expression vector, the *gcd* was cloned into pBBR1MCS-2 plasmid^1^. The recombinant plasmid was then introduced into *Pseudomonas* sp. Ps-12. Ps-12 carrying the recombinant plasmid pBBR1MCS-2-gcd was named Ps-12 (gcd). The phosphate-solubilizing ability, pH value, and *A*_600_ value of Ps-12 or Ps-12 (gcd) were detected in the NBRIP liquid medium.

### Determination of IAA and Ammonia of Ac-14

The Ac-14 strain was incubated overnight in LB medium under 180 r/min at 28 ± 2°C. The bacteria were then collected via centrifugation at 5,000 r/min for 2 min. After washing twice with LB liquid medium containing 5 mg/mL tryptophan, the suspension was inoculated into LB (containing 5 mg/mL tryptophan) liquid medium (*A*_600_ = 0.1). Thereafter, the bacteria were incubated at 28 ± 2°C and 180 r/min for 120 h. Two mL of the culture was collected every 24 h and centrifuged at 12,000 r/min for 1 min. Next, 1 mL of supernatant was mixed with 2 mL of Salkowski’s reagent (2% 0.5 M FeCl_3_ in 35% HClO_4_ solution) (Biswas et al., 2018). After reacting at room temperature under dark conditions for 30 min, indole acetic acid (IAA) was determined by measuring *A*_530_. The LB medium containing 5 mg/mL tryptophan was used as negative control.

The collected bacteria were washed twice with peptone water (peptone 10 g/L, NaCl 5 g/L, pH 7.0 ± 0.2). The biomass was adjusted to *A*_600_ = 0.1 using peptone water, and incubated under 180 r/min at 28 ± 2°C for 120 h. Culture samples were collected every 24 h and centrifuged at 12,000 r/min for 1 min. The supernatant was then reacted with Nessler’s reagent (Marques et al., 2010; Orhan, 2016) to determine ammonia by measuring *A*_420_. Peptone water was used as a negative control.

### Statistical Analysis

All the experiments concerning data comparisons were performed three times. Statistical analyses were performed using the S-N-K method of one-way ANOVA or independent samples *t*-test (95% confidence) with IBM SPSS Statistics 22.0 (SPSS Inc., Chicago, IL, United States). Values with different lowercases represented a significant difference at *P* < 0.05. ^∗^ or ^∗∗^ indicated significant difference for the *t*-test (*P* < 0.05 or *P* < 0.01).

## Results

### Isolation of PSB Strains From the Fruit Tree Rhizosphere Soil in KRD Regions

In total, 805 PSB strains were isolated from 18 fruit tree rhizosphere soil samples. Of these, 570 PSB strains were from KRD regions in Southwest China, and 235 PSB strains were from the NKRD regions (Table 1). Moreover, 89 strains (67 and 22 strains from KRD and NKRD, respectively) with different colony morphologies and larger SI values were screened after 16S rDNA sequencing. *Pseudomonas* sp. was present in both KRD and NKRD region soils, whereas *Acinetobacter* sp. and *Paraburkholderia* sp. only appeared in some soil samples of KRD regions (Table 1).

The phosphate-solubilizing ability of the 89 strains was evaluated by the Mo-blue method. Of these, 22 PSB strains could dissolve more than 300 mg/L of phosphate. Among these 22 strains, seven belonged to *Acinetobacter*, one belonged to *Paraburkholderia*, and 14 belonged to *Pseudomona*s (Supplementary Table S1). The *Acinetobacter* sp. Ac-14 isolated from the KRD region (GJ2, Yinjiang, Guizhou) revealed the highest phosphate-solubilizing ability.

One strain from each genus summarized in Supplementary Table S1 with the highest phosphate-solubilizing ability was selected for further study (Table 2). The colony morphology observation revealed that *Acinetobacter* sp. Ac-14 was white and circular with an SI of 2.37; *Paraburkholderia* sp. Pa-3 was white in the middle and translucent outside with an irregular edge and SI of 2.29; *Pseudomonas* sp. Ps-12 strain was yellow and circular with an SI of 1.66 (Supplementary Figure S1). The three strains were rod-shaped and gram-negative bacteria but were different sizes (Supplementary Figure S1). Phylogenetic tree by 16S rDNA sequencing confirmed that the three strains belonged to different groups (Supplementary Figure S2).

**TABLE 2:** The colony characteristics of *Acinetobacter* sp. Ac-14, *Paraburkholderia* sp. Pa-3, and *Pseudomonas* sp. Ps-12 from KRD regions in Southwest China.

Characteristics	Ac-14	Pa-3	Ps-12
Color	White	White in the middle with translucent around the outside	Yellow
Surface	Glossy	Moist	Glossy
Shape	Circular	Irregular edge	Circular
Uplift/shape	Raised	Uneven	Flat
Margin	Smooth	Smooth	Smooth
SI value	2.37 ± 1.09	2.29 ± 0.39	1.66 ± 0.94
Cell size (μm)	(0.58 ± 0.05) × (1.03 ± 0.16)	(0.49 ± 0.04) × (1.99 ± 0.17)	(0.54 ± 0.06) × (1.66 ± 0.19)
Gram character	Negative	Negative	Negative
Genus (by 16S rDNA)	*Acinetobacter*	*Paraburkholderia*	*Pseudomonas*

### PSB Strains, Particularly Ac-14, Had Sustained and Stable Phosphate-Solubilizing Ability Even Under CaCO_3_ Condition

**FIGURE 1:**
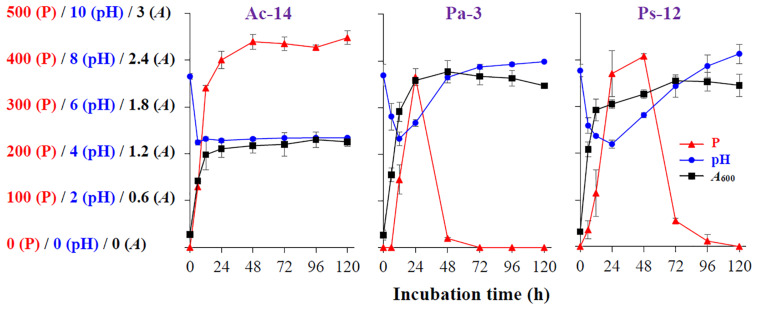
The time-curve of the soluble P content (P, mg/L), pH value (pH), and *A*_600_ value (*A*) of *Acinetobacter* sp. Ac-14, *Paraburkholderia* sp. Pa-3, and *Pseudomonas* sp. Ps-12.

To assess the phosphate-solubilizing characteristics of the isolated strains, their phosphate-solubilizing ability in liquid NBRIP medium, as well as the pH and bacterial *A*_600_ values, were determined. As illustrated in Figure 1, for Ac-14, the soluble P concentration in the medium rapidly elevated within 12 h, and then slowly increased between 12 and 48 h, and was further maintained at a high level (439–448 mg/L, 48–120 h). The pH value of the medium dropped rapidly from 7.3 to 4.5 within 6 h and was then maintained at a low level. The growth of biomass rapidly elevated to 1.2 within 12 h, and then slowly increased until it reached the stationary phase. Thus, the phosphate-solubilizing ability revealed a similar change as that of *A*_600_, whereas it revealed an inverse relation with the pH value. Furthermore, the lowest pH value appeared earlier than that of the maximum value of soluble P content and *A*_600_.

For Pa-3 (Figure 1), the soluble P concentration in the medium rapidly increased and further decreased, with the highest value (365 mg/L) at 24 h; moreover, soluble P could not be detected at 72 h. In contrast, the pH value rapidly decreased within 12 h and then increased. The growth of biomass rapidly elevated within 12 h, then increased slowly, and then decreased slowly. The phosphate-solubilizing ability, pH value, and growth of biomass of Ps-12 revealed a similar change tendency to that of Pa-3 (Figure 1).

**FIGURE 2:**
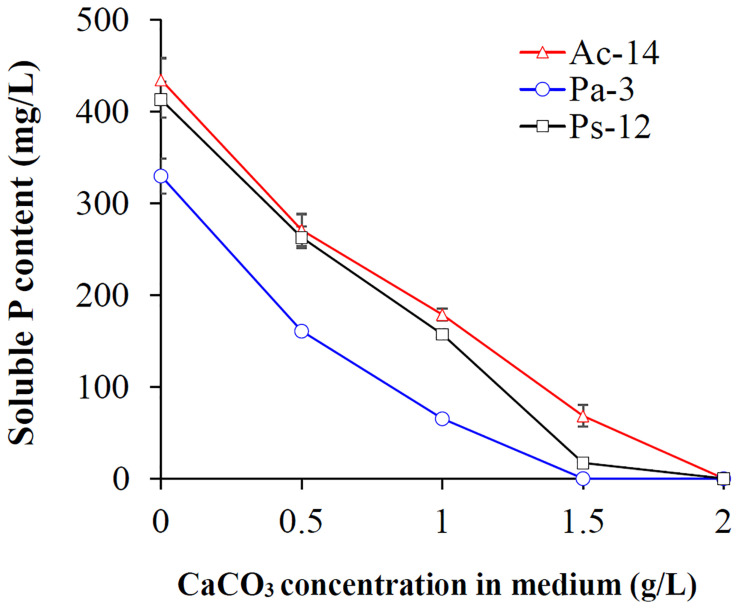
Effect of different concentrations of CaCO_3_ on the phosphate-solubilizing ability of strains Ac-14, Pa-3, and Ps-12.

**FIGURE 3:**
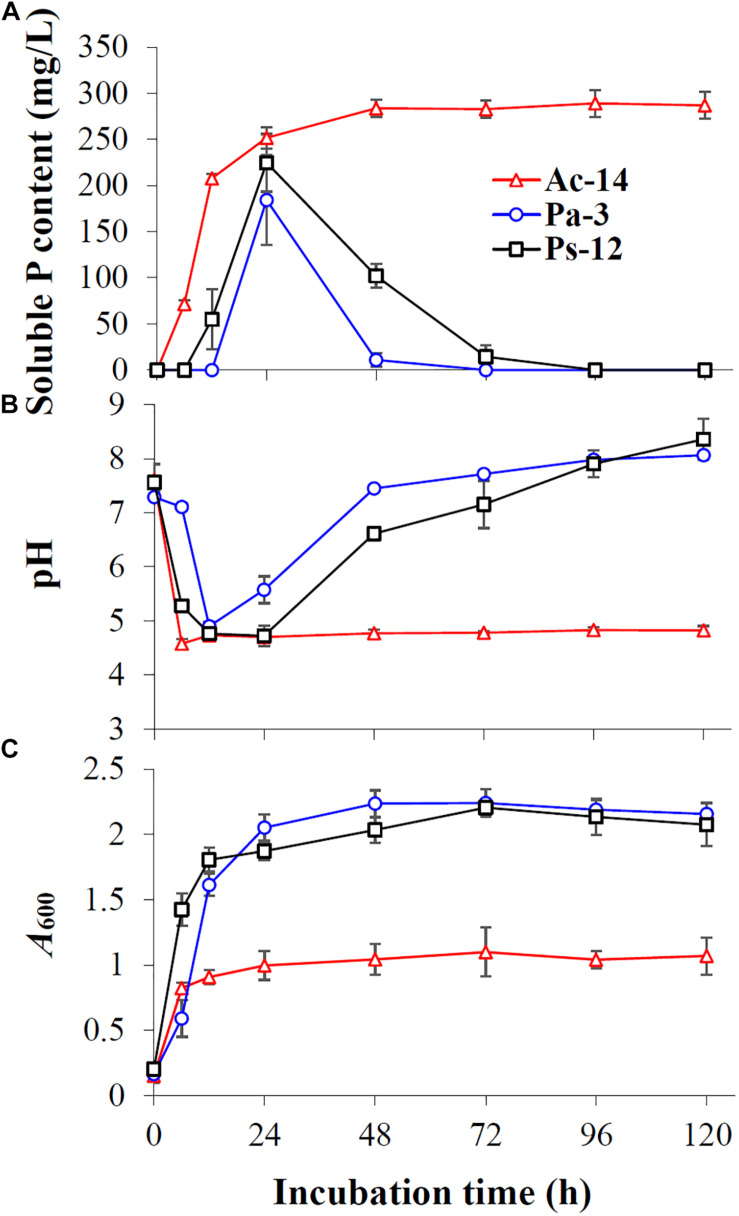
The time-curve of **(A)** the soluble P content, **(B)** pH value, and **(C)** *A*_600_ value of the strains Ac-14, Pa-3, and Ps-12 in NBRIP medium containing 0.5 g/L CaCO_3_.

As rocky desertification mainly occurs in carbonate rock areas, we studied the phosphate-solubilizing ability of the isolated PSB strains under CaCO_3_ condition (Figure 2). CaCO_3_ markedly decreased the phosphate-solubilizing ability of the strains, and its effect was increased as concentration increased. At a concentration of 2 g/L, soluble P could not be detected in the supernatant; however, Ac-14 still had a higher phosphate-solubilizing ability than Pa-3 and Ps-12 under each CaCO_3_ concentration condition. Under 0.5 g/L CaCO_3_ (Figure 3), the changes in the phosphate-solubilizing ability, pH value, and *A*_600_ value of the three PSB strains were similar to those without CaCO_3_, except for the lower maximum phosphate-solubilizing abilities than those without CaCO_3_ (Figure 1). The effect of CaCO_3_ on phosphate-solubilizing ability was limited in liquid medium, but not in agar-solidified medium (Supplementary Figure S3 and Supplementary Table S2).

Moreover, the effect of several insoluble phosphate sources on the phosphate-solubilizing ability of the aforementioned three PSB strains was studied. When Ca_3_(PO_4_)_2_ was substituted with equal amounts of AlPO_4_ or FePO_4_ (data not shown) in liquid NBRIP medium, soluble P could not be detected in the medium inoculated with Ac-14, Pa-3, and Ps-12, thus indicating that the three strains can only dissolve Ca_3_(PO_4_)_2_.

### Inoculation of Ac-14 to Roots of *Arabidopsis thaliana* Seedlings Promoted Vegetative Growth

**FIGURE 4:**
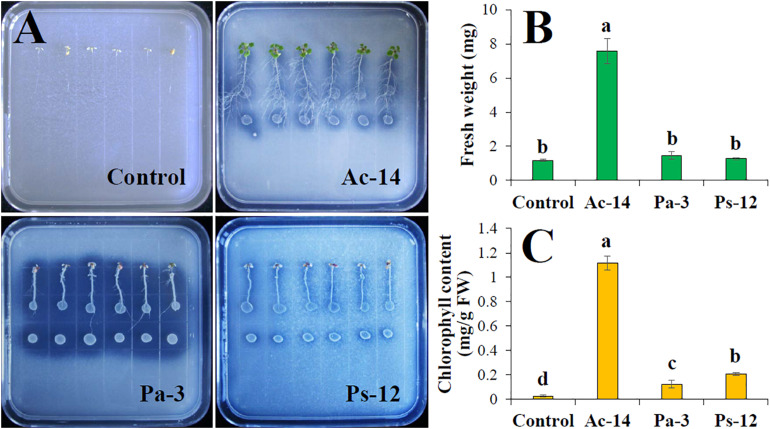
Effects of the strains Ac-14, Pa-3, and Ps-12 on the growth of *Arabidopsis thaliana* seedlings in NBRIP agar-solidified medium (cocultivation for 14 days). **(A)** The growth status, **(B)** fresh weight per plant, and **(C)** chlorophyll content of *A. thaliana*. Different lowercase letters indicated statistically significant differences (*P* < 0.05).

Since Ac-14, Pa-3, and Ps-12 had a strong ability to dissolve phosphate, their effect on plant growth needs to be explored. Therefore, we inoculated the strains in the roots of *A. thaliana* seedlings and observed the growth status. When Ac-14 was cocultured for 7 days, the number of lateral roots, fresh weight, and chlorophyll content began to increase; however, no change was observed in the primary root length (Supplementary Figures S4A-D). On the 14th day, the seedlings inoculated with Ac-14 grew vigorously and had developed lateral roots (Figure 4A). The fresh weight was increased 6.5-fold, and the chlorophyll content increased 46.5-fold (Figures 4B,C), compared to that without inoculation, thus indicating that Ac-14 could remarkably promote the growth of *A. thaliana* seedlings. Pa-3 and Ps-12 were also effective but had a lesser impact on the growth of *A. thaliana* seedlings compared to that of Ac-14.

**FIGURE 5:**
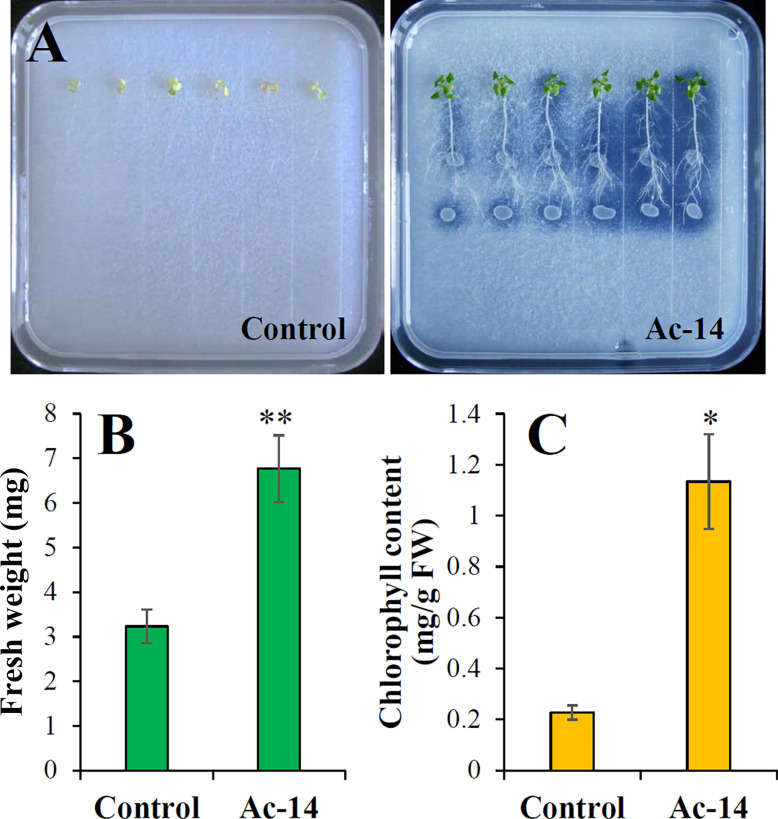
Effect of *Acinetobacter* sp. Ac-14 on the growth of *Arabidopsis thaliana* seedlings in NBRIP agar-solidified medium containing 0.5 g/L CaCO_3_ (cocultivation for 14 days). **(A)** The growth status, **(B)** fresh weight per plant, and **(C)** chlorophyll content of *A. thaliana*. *P < 0.05; **P < 0.01.

We further assessed the effect of Ac-14 on the growth of *A. thaliana* seedlings in a medium containing 0.5 g/L CaCO_3_. The seedlings inoculated with Ac-14 presented remarkable growth when cocultured for 7 days compared to that without Ac-14 (Supplementary Figures S4E-H). On the 14th day, the fresh weight of the seedlings inoculated with Ac-14 increased 2.1-fold, while the chlorophyll content increased 5-fold, compared to that without inoculation (Figure 5). These results indicated that inoculation of Ac-14 in the roots of *Arabidopsis* seedlings could promote vegetative growth in the presence and absence of CaCO_3_.

### Ac-14 Produced 23 Types of Organic Acids, While Gluconic Acid and D-(-)-Quinic Acid Increased Mostly

To detect the metabolites of Ac-14 related to phosphate solubilization, we performed untargeted metabolomics (Supplementary Figure S5). In total, 752 metabolites were detected; of these, 294 metabolites were detected under LC-MS/MS (ESI-) scan model, whereas 458 metabolites were detected under LC-MS/MS (ESI+) scan model (see sheet “All metabolites” in Supplementary Excel S1). According to fold change ≥ 1.5 or FC ≤ 0.667, VIP > 1, and *P*-value < 0.05, 124 differential metabolites were identified in Ac-14 compared to that without inoculation; of these, 56 metabolites (37 metabolites were significantly increased and 19 metabolites were significantly decreased) were detected under ESI- model, and 68 metabolites (30 metabolites were significantly increased and 38 metabolites were significantly decreased) were detected under ESI+ model (Supplementary Figure S5; sheet “Differential metabolites” in Supplementary Excel S1). As organic acids were produced during bacterial phosphate solubilization, the differences in organic acids were further analyzed. Ac-14 could produce 23 types of organic acids (13 kinds of organic acids were increased under ESI- model and 10 kinds of organic acids were increased under ESI+ model) with the concentration increasing from 4.1-fold to 462.2-fold. Among these organic acids, gluconic acid (366-fold) and D-(-)-quinic acid were remarkably increased (462-fold) (Table 3). These results indicated that Ac-14 could produce abundant organic acids.

**TABLE 3:** Comparison of organic acids between cultures inoculated with *Acinetobacter* sp. Ac-14 and non-inoculation under LC-MS/MS.

Name	FC	*P*-value	VIP	ESI−/+
D-(-)-quinic acid	462.2	3.17E-05	4.7	+
Gluconic acid	366.0	3.13E-05	3.7	−
Indole-5-carboxylic acid (98%)	142.5	1.98E-04	3.1	−
D-galactonic acid	119.8	1.82E-05	3.7	+
Pyrophosphate	111.1	8.50E-06	3.0	−
(R)-lipoic acid	60.5	1.82E-04	3.2	+
Mesaconic acid	51.6	7.52E-04	2.5	−
2-methylsuccinic acid	40.5	1.75E-03	2.9	+
Indole-3-lactic acid	39.0	2.08E-05	2.3	−
2-hydroxy-2-methylbutanedioic acid	34.9	2.09E-03	2.2	−
6-phosphogluconic acid	32.6	8.44E-04	2.7	+
2-(2-hydroxy-3-methylbutanamido)-4-methylpentanoic acid	27.2	8.80E-05	2.5	+
Kojic acid	16.5	1.71E-03	2.2	+
Kinic acid	15.5	6.28E-04	1.7	−
Suberic acid	12.1	1.77E-03	1.6	−
2-(acetylamino)-4-(methylthio) butanoic acid	9.7	7.48E-04	1.4	−
Uric acid	9.1	8.48E-05	1.7	+
Elaidic acid	7.6	5.72E-03	1.3	−
2-ketoadipic acid	7.0	2.65E-02	1.3	−
4-oxododecanedioic acid	6.3	5.18E-04	1.4	+
D-α-Hydroxyglutaric acid	5.9	1.09E-02	1.1	−
3-[(methoxycarbonyl) amino]-2,2,3-trimethylbutanoic acid	5.3	3.82E-03	1.1	−
4-(2,3-dihydro-1,4-benzodioxin-6-yl) butanoic acid	4.1	5.49E-04	1.1	+

**FC, fold change; P-value from univariate analysis (t-test); VIP, variable importance in the projection (VIP) value of the first principal component of PLS-DA model.**

### Expression of Ac-14 *gcd* Gene Conferred Ps-12 With Sustained and Stable Phosphate-Solubilizing Ability

**FIGURE 6:**
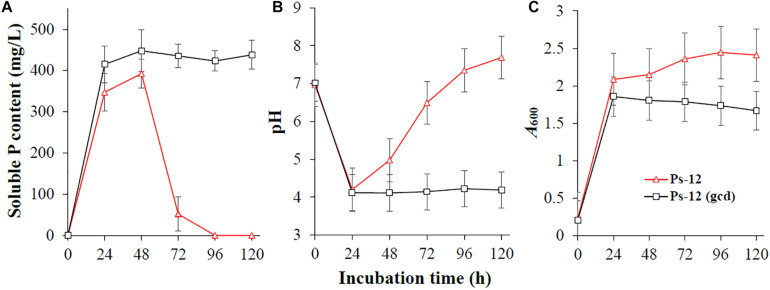
The effects of *Acinetobacter* sp. Ac-14 *gcd* gene on **(A)** the soluble P content, **(B)** pH value, and **(C)** *A*_600_ value of *Pseudomonas* sp. Ps-12.

To confirm the phosphate-solubilizing ability of *Acinetobacter* sp. Ac-14 related with gluconic acid, we cloned the Ac-14 *gcd* gene and expressed it in *Pseudomonas* sp. Ps-12. As illustrated in Figure 6, expression of *gcd* gene in Ps-12 could maintain a sustained and stable soluble P concentration (448 mg/L, 48 h; 438 mg/L, 120 h), whereas Ps-12 without *gcd* revealed a maximum soluble P level at 48 h (393 mg/L), then gradually decreased, and was further undetected at 120 h. As a result, Ps-12 with *gcd* could maintain a lower pH (4.1–4.2) during the experiment period; however, that without *gcd* presented decreased pH within 24 h, which then gradually increased. Ps-12 with *gcd* had a lower biomass than that without *gcd*. These results confirmed that the production of gluconic acid is an important mechanism conferred on bacteria with sustained and stable phosphate solubilization.

### Phosphate Solubilization of Ac-14 Did Not Occur via NH_4_^+^ Assimilation

NH_4_^+^ assimilation can release protons and results in a decreased pH. Whether the sustained and stable phosphate-solubilizing ability of Ac-14 is related to NH_4_^+^ assimilation remains unclear. Hence, the phosphate-solubilizing ability, pH value, and *A*_600_ value under different nitrogen sources [(NH_4_)_2_SO_4_, NH_4_Cl, NaNO_3_, or KNO_3_] were determined. No significant difference was observed under different nitrogen sources (Supplementary Figure S6), indicating that the phosphate-solubilizing ability of Ac-14 did not occur due to NH_4_^+^ assimilation.

### Ac-14 Produced IAA and Ammonia

**FIGURE 7:**
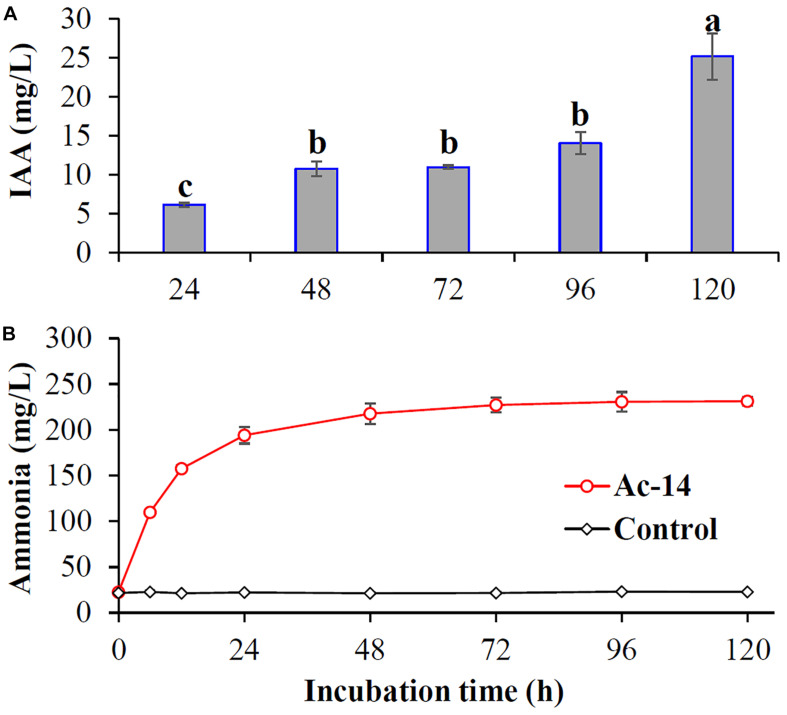
The content of **(A)** IAA and **(B)** ammonia produced by *Acinetobacter* sp. Ac-14. Different lowercase letters indicated statistically significant differences (*P* < 0.05).

Whether other mechanisms besides phosphate solubilization are involved in the growth promoting effect of Ac-14 remains unclear. Hence, the plant growth promoting traits (like production of IAA and ammonia) for Ac-14 were detected. IAA was increased with the incubation time, and the concentration reached 25.1 mg/L at 120 h. Furthermore, ammonia was also increased and then maintained at a stable level during the incubation period, and the concentration was 231 mg/L at 120 h (Figure 7).

### Ac-14 Grew in a Wide Range of pH

As Ac-14 can produce organic acids, the environmental pH range it can withstand remains unclear. Hence, the growth status of Ac-14 under different pH values was explored (Supplementary Figure S7). The strain grew best at pH 5–7, and the *A*_600_ value was approximately 2.3. At pH 8–9, *A*_600_ value was approximately 2.2. When pH was 4.5 or 10, *A*_600_ value was about 2.1. When pH was 11, the growth of the strain was severely affected and *A*_600_ value was only 0.12. When pH was less than 4 or greater than 12, the bacteria could not grow. This result indicated that Ac-14 could grow at various pH ranges.

## Discussion

### *Acinetobacter* sp. Ac-14 With Phosphate-Solubilizing Ability Was Successfully Isolated From KRD Soil

P is an important macro-element in plant nutrition. PSB can transform insoluble P into an available form in the soil and has immense applications in ecoagriculture. Due to the limitations of microbial ecological adaptability, the utilization of native microorganisms to develop biological fertilizer has obvious advantages (Wang et al., 2018). Therefore, it is important to isolate PSB that efficiently dissolves phosphate and promotes plant growth from KRD soil samples. In this study, 805 PSB strains were isolated from the rhizosphere soil of fruit trees. Of these, 570 were from 15 soil samples in the KRD regions in Southwest China, while 235 were isolated from three soil samples from NKRD regions. Further analysis revealed that *Acinetobacter* sp. and *Paraburkholderia* sp. were uniquely distributed in fruit tree rhizosphere soil of KRD regions. *Acinetobacter* sp. Ac-14 could efficiently dissolve phosphate and promote plant growth. Therefore, Ac-14 has a potential application in ecological restoration and development in KRD regions.

Previous studies reported that *Pseudomonas*, *Bacillus*, and *Rhizobium* are the most efficient phosphate solubilizers (Rodríguez and Fraga, 1999). In recent years, *Acinetobacter* was reported to have a high phosphate-solubilizing ability. *Acinetobacter calcoaceticus* YC-5a has a strong ability for solubilizing insoluble phosphate by producing organic acid and some plant growth-promoting factors such as IAA and siderophores. Moreover, this bacterium exhibits strong resistances to lead and antibiotics (Ren et al., 2013). *Acinetobacter* sp. YU-SS-SB-29, isolated from monazite sand, exhibits high phosphate solubilization and tolerance to uranium (Sowmya et al., 2014). Furthermore, *Acinetobacter* was also reported to effectively degrade toxic organic compounds (Li et al., 2020). In this study, *Acinetobacter* sp. Ac-14 isolated from fruit tree rhizosphere soil of KRD region had a high phosphate-solubilizing ability, further confirming that *Acinetobacter* can dissolve phosphate. Whether the bacterium is capable of resisting metal and degrading toxic organic compounds needs to be further studied.

### Production of Gluconic Acid Is the Core Mechanism of Ac-14 During Phosphate Solubilization

The mechanisms of PSB involved in phosphate solubilization are complex, and various mechanisms have been proposed; of these, low pH is common. In the present study, Ac-14 revealed efficient, sustained, and stable phosphate-solubilizing ability, with the pH value was maintained at a low level. These findings are in accordance with previous studies (Lin et al., 2006; Wang et al., 2019). For Pa-3 and Ps-12, the phosphate-solubilizing ability was initially increased and then decreased with the incubation time, whereas the pH value was initially decreased and then increased. Therefore, the phosphate solubilization mechanism of these three strains may relate to pH changes; however, the changes occurring in the three strains are not exactly the same, thereby indicating differences in the mechanism of phosphate solubilization. Similar changes of pH and phosphate solubilization to that of Pa-3 and Ps-12 were reported previously (Teng et al., 2019). The maximum *A*_600_ of Ac-14 was remarkably lower than that of Pa-3 and Ps-12, which was presumably because low pH in the environment inhibited bacterial proliferation. In the NBRIP liquid medium containing CaCO_3_, the phosphate-solubilizing ability of the three PSB strains was decreased. This may occur because abundant Ca^2+^ combined with soluble P to form insoluble phosphate; however, the changed tendencies of phosphate-solubilizing ability, pH value, and *A*_600_ under CaCO_3_ condition were almost similar to those without CaCO_3_. Interestingly, we also found that inhibition of CaCO_3_ did not affect the phosphate-solubilizing ability in the agar-solidified medium, which might be due to the poor mobility of CaCO_3_ in this medium. Therefore, application of PSB strains as biological fertilizers in the soil may not be affected by CaCO_3_ in KRD soils.

Furthermore, it was reported that low pH could occur due to production of organic acids and assimilation of NH_4_^+^ (Rodríguez and Fraga, 1999; Sharma et al., 2013; Owen et al., 2015; Zhu et al., 2018). In this study, metabolomics analysis revealed that Ac-14 could substantially increase 23 types of organic acids; of these, gluconic acid, one of the most common organic acid metabolites (Lin et al., 2006; Rodríguez et al., 2006; Ludueña et al., 2018), was highly increased. It was reported that gram-negative bacteria produce gluconic acid during the extracellular oxidation of glucose through GCD (Liu et al., 1992; Goldstein, 1995), the lowered pH value and changed reduction potential are considered reasons for the dissolution of tricalcium phosphate (Lin et al., 2006). As a Gram-negative bacterium, Ac-14 may lower the pH value by producing gluconic acid, after excluding the possibility of NH_4_^+^ assimilation. This was further demonstrated by the genetic engineering approach that suggested that *Pseudomonas* sp. Ps-12 expressing Ac-14 *gcd* gene could maintain sustained and stable phosphate-solubilizing ability. Besides the common gluconic acid, D-(-)-quinic acid was also highly increased. D-(-)-quinic acid is an efficient low molecular mass organic acid chelator. It can bind with metal ions (Menelaou et al., 2009) and acts as a metabolite with an antioxidant function (Gargallo-Garriga et al., 2015), which may confer the bacterium with tolerance and/or metal-degrading ability. The exact role of D-(-)-quinic acid in phosphate solubilization, and other mechanisms, needs to be further studied.

### Ac-14 Promotes the Growth of *A. thaliana* Seedling by Phosphate Solubilization and Produces IAA and Ammonia

It was reported that PSB could promote the growth of numerous plants, such as *Lolium perenne*, *Zea mays*, *Vigna radiata*, Chinese cabbage, etc. (Owen et al., 2015; Biswas et al., 2018; Wang et al., 2019). In general, PSB enhances the root growth of plants and the yield of crops by increasing P availability (Sharon et al., 2016; Ku et al., 2018). In this study, we explored the effects of Ac-14, Pa-3, and Ps-12 on the growth of *A. thaliana* seedlings. The number of lateral roots, fresh weight, and chlorophyll content were remarkably increased when *A. thaliana* was inoculated with Ac-14, whereas the leaves of *A. thaliana* without inoculation gradually turned white and died. However, not all PSB strains can promote plant growth (Bashan et al., 2013). In this study, Pa-3 or Ps-12 could not significantly promote *A. thaliana* seeding growth. Furthermore, the process of insoluble P-solubilization is affected by several factors including soil type, nutritional richness of the soil, pH, moisture, and others (Rodríguez and Fraga, 1999; Chen et al., 2006; Alori et al., 2017; Wei et al., 2018). On the other hand, PSB would compete with other soil microflora (Wei et al., 2018). Whether Ac-14 could successfully colonize in KRD regions soil and exhibit phosphate-solubilizing ability and growth-promoting activities needs further research.

In this study, we revealed that the Ac-14 strain could produce certain growth-promoting substances such as IAA and ammonia. As a phytohormone, IAA promotes root growth to achieve a large surface area that facilitates nutrient absorption from the soil (Davies, 2010; Biswas et al., 2018; Wang et al., 2019). It was reported that some PSB strains, such as *A. calcoaceticus* YC-5a, *Bacillus* sp., *Staphylococcus* sp., and *Serratia* sp. could also produce IAA (Ren et al., 2013; Biswas et al., 2018; Ludueña et al., 2018). Ammonia is a nitrogen source that can be taken up by plants and could promote plant growth. It was reported that all test strains isolated from plant root nodules of different rhizospheric soils in the vicinity of Aligarh could produce ammonia (Ahmad et al., 2008). Three PSB strains isolated from earthworms (*Metaphire posthuma*) could also produce ammonia (Biswas et al., 2018). Therefore, Ac-14 promoting *A. thaliana* seedling growth might also relate to the secretion of IAA and ammonia.

## Conclusion

In this study, we isolated PSB strains from the fruit tree rhizosphere soil in the KRD regions in Southwest China. A novel PSB strain, *Acinetobacter* sp. Ac-14, presented efficient, sustained, and stable phosphate-solubilizing ability, and could also promote plant growth. The strain dissolved insoluble phosphate by producing 23 types of organic acids; of these, gluconic acid played an important role in the solubilization process. Ac-14 could also produce plant growth-promoting substances such as IAA and ammonia. Therefore, Ac-14 may adapt to the high calcium environment in KRD regions and has the potential to be utilized in improving the fertility of KRD regions.

## Data Availability Statement

The datasets presented in this study can be found in online repositories. The names of the repository/repositories and accession number(s) can be found in the article/Supplementary Material.

## Author Contributions

XC and DC designed the experiments. JX, ZY, GW, WX, and CL performed the experiments. JX, ZY, and DC analyzed the data. JX, XC, and DC wrote the manuscript. All authors discussed the results and implications and commented on the manuscript at all stages.

## Conflict of Interest

The authors declare that the research was conducted in the absence of any commercial or financial relationships that could be construed as a potential conflict of interest.

## Footnotes

1 http://www.addgene.org/85168/
