# Supplementary material for: A Bacterium Isolated From Soil in a Karst Rocky Desertification Region Has Efficient Phosphate-Solubilizing and Plant Growth-Promoting Ability
Source: Front Microbiol. 2021 Feb 1;11:625450. doi: 10.3389/fmicb.2020.625450 (PMC7882523; doi:10.3389/fmicb.2020.625450)
Supplement: Supplementary file 1 [file Data_Sheet_1.pdf]

Supplementary Information for:

A Bacterium Isolated from Soil in a Karst Rocky Desertification Region Has Efficient Phosphate-Solubilizing and Plant Growth-Promoting Ability

***Jinge Xie*[1], *Zongqiang Yan*[1], *Guifen Wang*[1], *Wenzhi Xue*[2], *Cong Li*[1], *Xiwen Chen*[2, *], *Defu Chen*[1, *]**

---

[1] *Department of Genetics and Cell Biology, College of Life Sciences, Nankai University, Tianjin, China*

[2] *Department of Biochemistry and Molecular Biology, College of Life Sciences, Nankai University, Tianjin, China*

* **Correspondence:**

Xiwen Chen: xiwenchen@nankai.edu.cn

Defu Chen: chendefu@nankai.edu.cn

This file includes:

Supplementary Materials and methods

Supplementary Table 1-2

Supplementary Figure1-7

Supplementary Excel 1

Supplementary Materials and Methods

Collection of Soil Samples and Isolation of PBS Strains

NBRIP medium: $Ca_3(PO_4)_2$ 5 g, glucose 10 g, $MgCl_2·6H_2O$ 5 g, $MgSO_4·7H_2O$ 0.25 g, KCl 0.2 g, and $(NH4)_2SO_4$ 0.1 g, dissolved in 1 L of distilled water.

Modified NBRIP medium: additionally added $AlPO_4$ 2.5 g/L and $FePO_4$ 2.5 g/L as the source of P, as well as the following nutrient substance (40 mL/L soil extract, microelements and organic matter).

The soil extract: 0.5 kg of unfertilized garden soil was taken, after removing debris and then sieved, the soil was placed in a sterile Erlenmeyer flask, and adding 1 L of sterile water. With the mouth of the bottle sealing with a breathable plug, the soil was heated in boiling water for 2 h in a water bath, filtered after cooling, made up to the volume into 1 L with sterile water and stored at 4°C.

The microelements: $ZnSO_4·7H_2O$ 8.82 mg, $MnCl_2·4H_2O$ 1.44 mg, $NaMoO_4·2H_2O$ 1.21 mg, $CuSO_4·5H_2O$ 1.57 mg, $CoCl_2·6H_2O$ 0.4 mg, H_3BO_3 11.42 mg, Na_2-EDTA·$2H_2O$ 50 mg, KOH 31 mg, $FeSO_4·7H_2O$ 4.98 mg and H_2SO_4 1 μL, dissolved in 1 L of distilled water.

The organic matter: vitamin B_1 0.1 mg, vitamin B_6 0.5 mg, niacin 0.5 mg, inositol 0.1 g and glycine 0.02 g, dissolved in 1 L of distilled water.

AT salt medium: glucose 10 g, KH_2PO_4 10.9 g, $(NH_4)_2SO_4$ 1 g, $MgSO_4·7H_2O$ 0.16 g, $FeSO_4·7H_2O$, 0.005 g, $CaCl_2·2H_2O$ 0.011 g and $MnCl_2·4H_2O$ 0.002 g, dissolved in 1 L of distilled water.

Untargeted Metabolomics of Ac-14

Liquid sample (100 μL) and prechilled methanol (400 μL) were mixed well by vortexing. The samples were incubated on ice for 5 min and then centrifuged at 15,000 r/min, 4°C for 5 min. Part of supernatant was diluted to final concentration containing 53% methanol by LC-MS

grade water. The samples were subsequently transferred to a fresh Eppendorf tube, and then centrifuged at 15,000 g, 4°C for 10 min. Finally, the supernatant was injected into the LC-MS/MS system for analysis.

LC-MS/MS analyses were performed using a Vanquish UHPLC system (Thermo Fisher Scientific, MA, USA) coupled with an Orbitrap Q Exactive series mass spectrometer (Thermo Fisher Scientific, MA, USA). Samples were injected into a Hyperil Gold column (100 × 2.1 mm, 1.9 μm) using a 16 min linear gradient at a flow rate of 0.2 mL/min. The eluents for the positive polarity mode were eluent A (0.1% formic acid in water) and eluent B (Methanol). The eluents for the negative polarity mode were eluent A (5 mM ammonium acetate, pH 9.0) and eluent B (Methanol). The solvent gradient was set as follows: 2% B, 1.5 min; 2-100% B, 12.0 min; 100% B, 14.0 min; 100-2% B, 14.1 min; 2% B, 17 min. Q Exactive series mass spectrometer was operated in positive/negative polarity mode with spray voltage of 3.2 kV, capillary temperature of 320°C, sheath gas flow rate of 35 arb and aux gas flow rate of 10 arb.

The raw data files generated by UHPLC-MS/MS were processed using the Compound Discoverer 3.1 (CD3.1, Thermo Fisher Scientific, MA, USA) to perform peak alignment, peak picking, and quantitation for each metabolite. The main parameters were set as follows: retention time tolerance, 0.2 minutes; actual mass tolerance, 5 ppm; signal intensity tolerance, 30%; signal/noise ratio, 3; and minimum intensity, 100,000. After that, peak intensities were normalized to the total spectral intensity. The normalized data was used to predict the molecular formula based on additive ions, molecular ion peaks and fragment ions. And then peaks were matched with the mzCloud (https://www.mzcloud.org/), mzVault and MassList database to obtain the accurate qualitative and relative quantitative results.

Principal components analysis (PCA) and partial least squares discriminant analysis (PLS-DA) were performed at metaX (a flexible and comprehensive software for processing metabolomics data). We applied univariate analysis (*t*-test) to calculate the statistical

significance (P-value). Variable importance in the projection (VIP) value of the first principal component of PLS-DA model. The metabolites with VIP > 1 and P-value < 0.05 and fold change ≥ 1.5 or FC ≤ 0.667 were considered to be differential metabolites. Volcano plots were used to filter metabolites of interest which based on $\log_2$ (FC) and $-\log_{10}$ (P-value) of metabolites.

Cloning and Expression of the *Acinetobacter* sp. Ac-14 *gcd*

The sequence of Ac-14 *gcd* gene as follow:

atgaatcaacctacttcaagatcaggtttaacgacttttaccgtaattattattgggttactggcgttattcctgttaattggaggtatttggctcgctacactaggc
ggttcaatttactacattatagctggagtattactcctcattgttgcatggcaactctacaagcgtgcttctactgctttgtgggtttatgctgcattaatgctaggaa
ctattatctggagtgtctgggaagttggaacagacttttgggcgcttgcaccacgtttagatattttaggtattcttggtttatggttattggttccggctgtaactcg
tggaatcaacaaccttggatcaagtaaagttgccttatcttcaactttagcaattgcaatcgtgttgatggtttattctatcttcaatgatccgcaagaaattaatggt
gaaatcaaaacacctcaaccagaaacagcccaagccgtgcctggtgttgctgaaagtgattggccagcttatggccgtactcaagcaggtgtgcgttattct
ccattgaaacagatcaatgaccaaaacgtaaaagacttgaaagttgcttggactttacgtactggcgatcttaagacagataacgattctggtgaaacgacca
atcaggttacaccgattaaaatcggtaataacatgtttatctgtacagctcaccagcagttaattgcgattgatcctgctacaggtaaagaaaaatggcgttttga
tccgaaacttaaaacggataaatcgttccagcatttaacttgtcgtggtgtgatgtactacgatgcaaacaatacaactgagtttgcaacgagtcttcaaagcaa
gaaatctagctctacacaatgtccacgtaaggtatttgtaccagtcaatgatggccgtttagtggctgtaaatgctgacactggtaaagcatgtactgactttggt
caaaatggtcaagtgaacttacaagagttcatgccatatgcttatccaggcggttataacccgacatctcctggtatcgtgactggttcaactgttgttattgctg
gttctgtaacagataactactcaaataaagagccatctggtgtgattcgtggatatgatgttaacactggtaaacttctttgggtatttgatactggcgcagcaga
tccaaatgcaatgccgggcgaaggtactacttttgttcataactcaccaaatgcgtgggcacctttagcatacgatgccaaacttgatatcgtttatgtaccaac
aggtgtaggtacaccagacatctggggtggtgaccgtaccgagctgaaagagcgttatgcaaactcaatgttagcgattaatgcttctactggtaaattagtgt
ggaacttccaaacaacccatcacgatttatgggatatggacgtaccatcacaaccatctttagctgatatcaaaaacaaagctggccaaactgttcctgcaatc
tatgtattgacgaaaacaggtaatgcctttgtgcttgatcgccgtaatggtcaaccgattgttcctgtaactgagaaaccggttccacaaacagttaaacgtgga
ccacaaactaaaggtgagttctattcaaaaactcagccattctctgacttgaacttggcgccacaagataaattgactgataaagacatgtggggtgccactat
gcttgatcagctcatgtgtcgtgtatctttcaaacgtctaaattacgatggtatttatacgccaccatctgaaaacggtactttagttttccctggtaacttaggtgtg
tttgaatggggtggtatgtcagttaaccctgatcgtcaggttgctgtaatgaacccgattggtctgccattcgtcagtcgtttaattcctgctgatccaaaccgtg
cacaaactgcaaaaggtgcgggaactgagcagggtgtacaaccgatgtacggcgtaccgtatggtgttgaaattagcgcattcttatctccgcttggtttacc
ttgtaaacaaccggcttggggctatgtagctggcgttgatttgaaaactcatgaagtggtatggaaaaaacgtattggtacaattcgtgacagcttgccgaact
tgttccagttaccagctgtgaaaattggtgtgcctggtttaggtggttcaatttctactgccggtaatgtcatgtttgttggtgcaactcaagataactacttacgtg
cgtttaacgttactaacggtaagaaactttgggaagcgcgtttaccagcaggtggacaagcaacaccaatgacttatgaaatcaatggtaagcaatatgttgt
aatcatggctggtggtcatggttcatttggtacgaaaatgggcgactatttagtggcttatgccttaccagataacaaataa

Supplementary Tables

Table S1 | The information of the isolated PSB strains with phosphate-solubilizing ability higher than 300 mg/L in liquid NBRIP medium.

Table S2 | The SI value of *Acinetobacter* sp. Ac-14, *Paraburkholderia* sp. Pa-3, and *Pseudomonas* sp. Ps-12 under different concentrations of $CaCO_3$.

Table S1 | The information of the isolated PSB strains with phosphate-solubilizing ability higher than 300 mg/L in liquid NBRIP medium.

Soil code	Strain code	Soluble P content (mg/L)	PSB species by 16S rDNA
GJ2	**Ac-14 (or 2GJ2-R-14)**	**441 ± 14**	*Acinetobacter* sp.
YY1	2YY1-RS-1	405 ± 17	
GJ2	2GJ2-R-2	394 ± 29	
GJ2	2GJ2-RS-5	392 ± 35	
GJ2	2GJ2-RS-2	381 ± 11	
GJ2	2GJ2-RS-3	369 ± 15	
GJ2	162GJ2-R-7	365 ± 11	
XB1	**Pa-3 (or 3XB1-R-3)**	**330 ± 19**	*Paraburkholderia* sp.
NY1	**Ps-12 (or 2NY1-R-12)**	**413 ± 20**	*Pseudomonas* sp.
NY1	2NY1-R-4	408 ± 12	
HT1	3HT1-RS-1	393 ± 10	
GY1	2GY1-RS-17	381 ± 20	
CX3	2CX3-RS-1	380 ± 17	
XQ1	3XQ1-RS-4	367 ± 20	
EJ1	2EJ1-RS-8	365 ± 18	
EJ1	2EJ1-R-6	363 ± 16	
HZ1	3HZ1-R-4	362 ± 13	
CX1	2CX1-NR-15	354 ± 3	
CX2	1CX2-R-8	335 ± 15	
CX1	2CX1-NR-3	333 ± 16	
GJ3	1GJ3-RS-30	325 ± 15	
HZ1	3HZ1-R-1	321 ± 17	

Note: mean ± standard deviation (n = 3).

Table S2 | The SI value of *Acinetobacter* sp. Ac-14, *Paraburkholderia* sp. Pa-3, and *Pseudomonas* sp. Ps-12 under different concentrations of $CaCO_3$.

**$CaCO_3$ concentration (g/L)**	**Ac-14**	**Pa-3**	**Ps-12**
0	1.75 ± 0.36	2.42 ± 0.39	1.25 ± 0.09
0.5	1.62 ± 0.26	2.28 ± 0.43	1.27 ± 0.15
1.0	1.74 ± 0.41	1.66 ± 0.57	/
1.5	1.87 ± 0.38	1.78 ± 0.40	/
2.0	2.00 ± 0.38	2.24 ± 0.49	/

/, not determined.

Supplementary Figures

Figure S1 | The halos of colony, cell morphology revealed by scanning electron microscopy, and gram staining of *Acinetobacter* sp. Ac-14, *Paraburkholderia* sp. Pa-3, and *Pseudomonas* sp. Ps-12.

Figure S2 | Phylogenetic tree based on the 16S rDNA sequences of *Acinetobacter* sp. Ac-14, *Paraburkholderia* sp. Pa-3, and *Pseudomonas* sp. Ps-12.

Figure S3 | The phosphate-solubilizing halo zone of the strains Ac-14, Pa-3, and Ps-12 under different concentrations of $CaCO_3$.

Figure S4 | Effects of PSB strains on the growth of *Arabidopsis thaliana* seedlings after cocultivation for 7 days.

Figure S5 | Multivariate statistical analysis of untargeted metabolomics data obtained using the LC-MS/MS approach.

Figure S6 | The phosphate-solubilizing ability, pH value and A_{600} value of *Acinetobacter* sp. Ac-14 under different nitrogen sources.

Figure S7 | Effect of different pH on the growth of *Acinetobacter* sp. Ac-14.

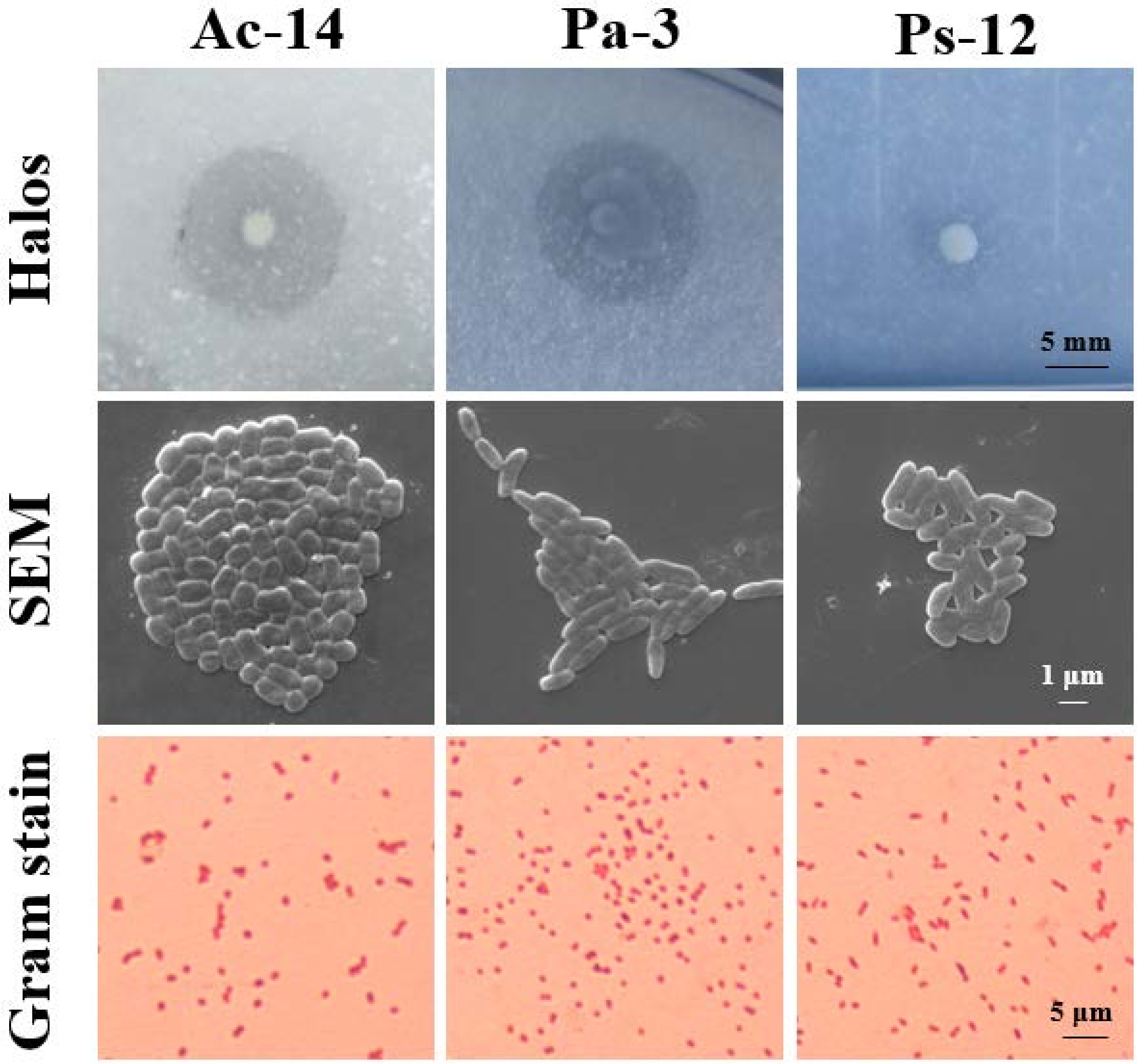

Figure S1 | The halos of colony, cell morphology revealed by scanning electron microscopy, and gram staining of *Acinetobacter* sp. Ac-14, *Paraburkholderia* sp. Pa-3, and *Pseudomonas* sp. Ps-12.

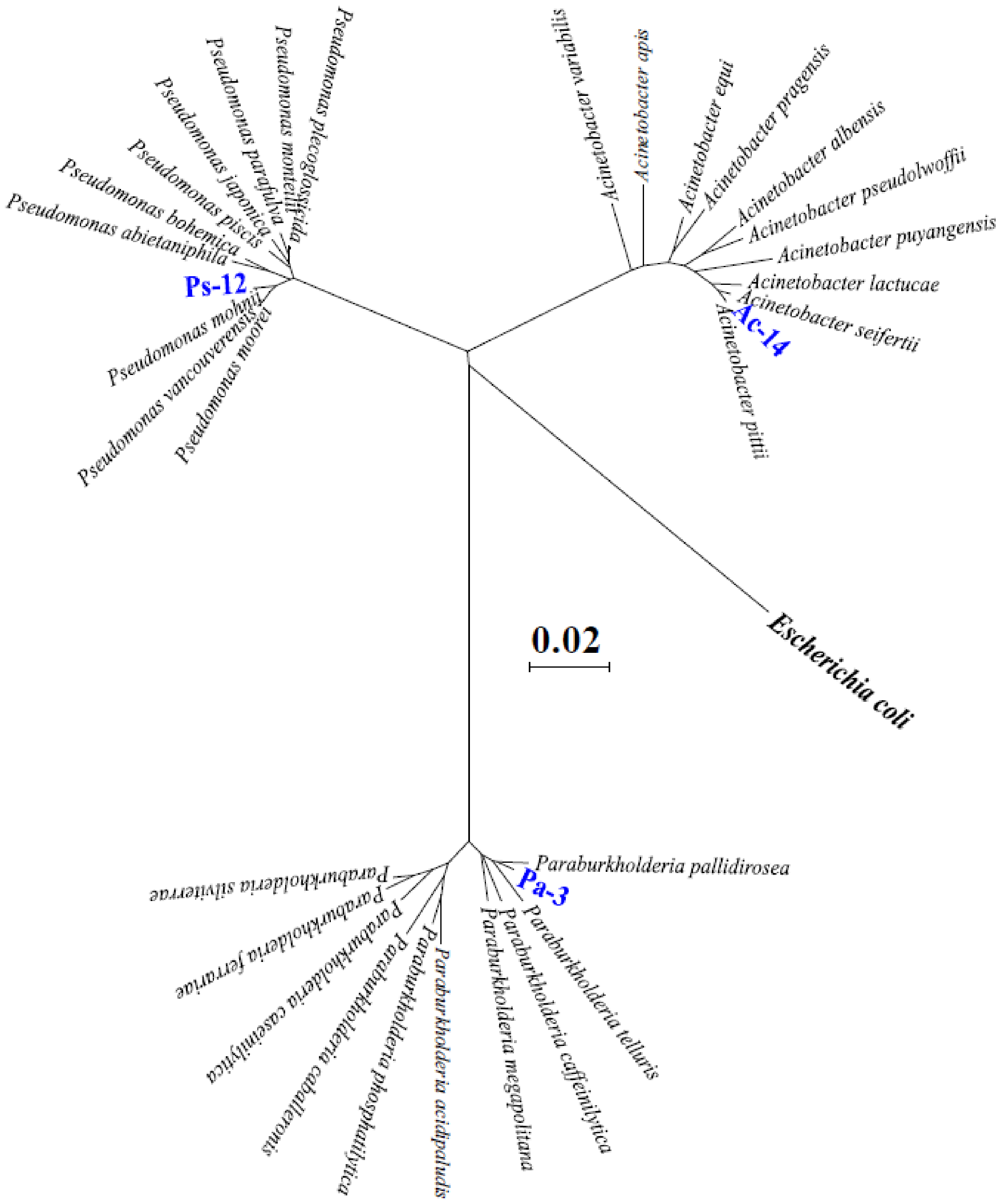

Figure S2 | Phylogenetic tree based on the 16S rDNA sequences of *Acinetobacter* sp. Ac-14, *Paraburkholderia* sp. Pa-3, and *Pseudomonas* sp. Ps-12. *Escheruchia coil* K12 was outgroup. The type strains, *Acinetobacter albensis* (KR611798.1), *Acinetobacter apis* (JX402203.1), *Acinetobacter equi* (KC494698.1), *Acinetobacter lactucae* (KU921101.1), *Acinetobacter pittii* (FJ694758.1), *Acinetobacter pragensis* (KX014586.1), *Acinetobacter pseudolwoffii*

(PHRG01000001.1), *Acinetobacter puyangensis* (JN664255), *Acinetobacter seifertii* (FJ860878.1), *Acinetobacter variabilis* (KP278590.1), *Paraburkholderia acidipaludis* (AB537485), *Paraburkholderia caballeroni* (EF139186) *Paraburkholderia caffeinilytica* (KT607985), *Paraburkholderia caseinilytica* (MF950896), *Paraburkholderia ferrariae* (AB537487), *Paraburkholderia megapolitana* (AM489502), *Paraburkholderia pallidirosea* (KP938221), *Paraburkholderia phosphatilytica* (MH393406), *Paraburkholderia silviterrae* (MF979829), *Paraburkholderia telluris* (MF979827), *Pseudomonas abietaniphila* (AJ011504.1) *Pseudomonas bohemica* (MG190030.1), *Pseudomonas japonica* (AB681920.1), *Pseudomonas mohnii* (AM293567.1), *Pseudomonas monteilii* (AB681966.1), *Pseudomonas moorei* (AM293566.1), *Pseudomonas parafulva* (AB681092.1), *Pseudomonas plecoglossicida* (AB681970.1), *Pseudomonas piscis* (LC500601.1), *Pseudomonas vancouverensis* (AJ011507.1). The 16S rDNA sequences of the isolated strains were submitted to GenBank under accession numbers MW011756 for Ac-14, MW011757 for Pa-3 and MW011758 for Ps-12. The scale bar indicates 0.02 substitutions per nucleotide position.

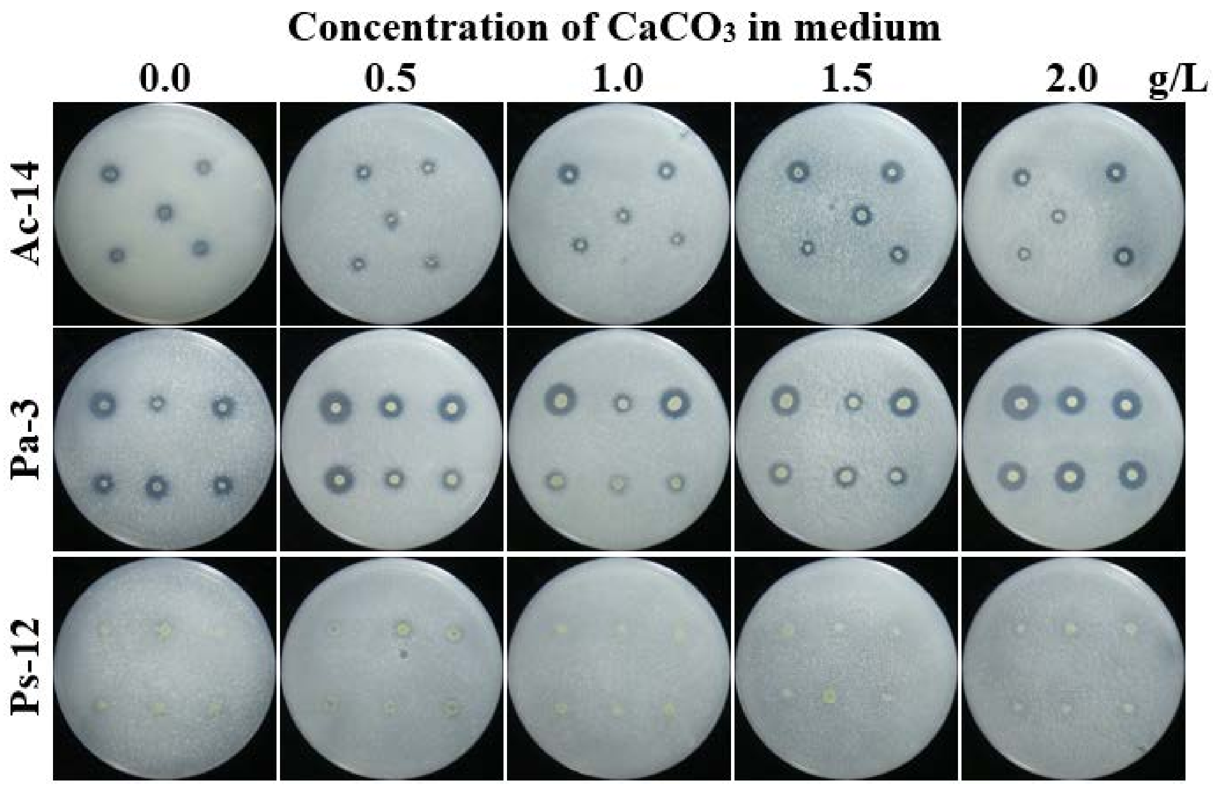

Figure S3 | The phosphate-solubilizing halos of the strains Ac-14, Pa-3, and Ps-12 under different concentrations of $CaCO_3$.

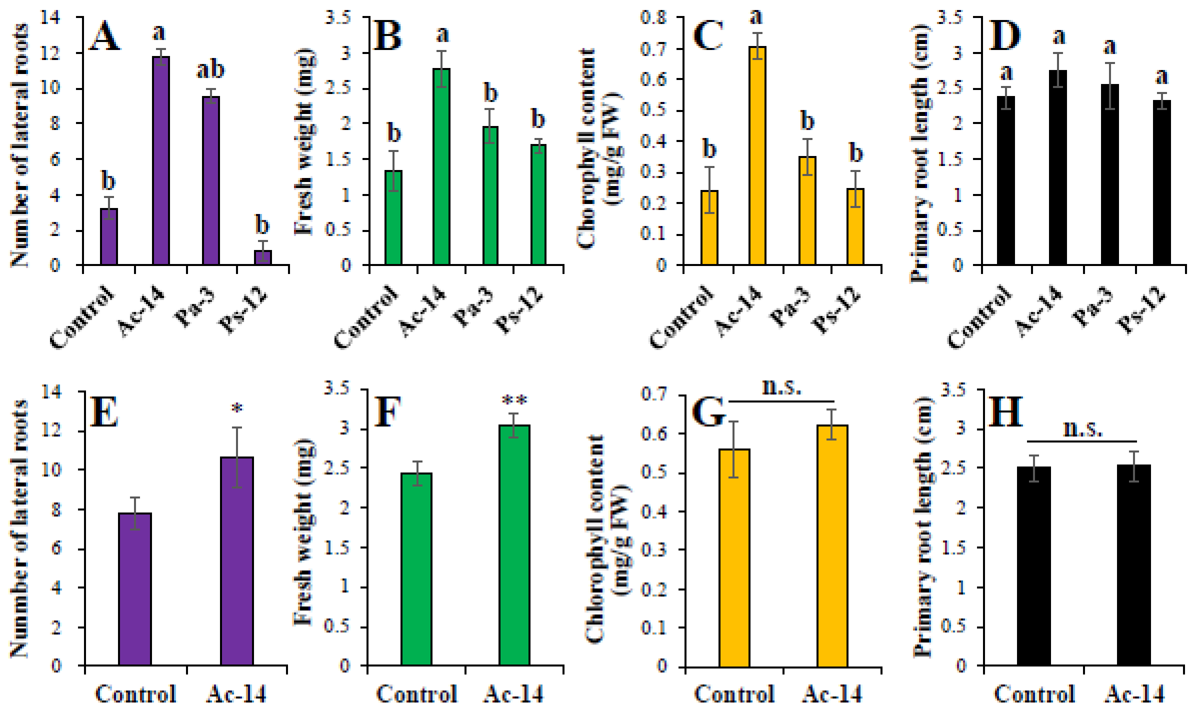

Figure S4 | Effects of PSB strains on the growth of *Arabidopsis thaliana* seedlings after cocultivation for 7 days. (A-D) *A. thaliana* seedlings cocultured with the strains Ac-14, Pa-3, and Ps-12 in NBRIP agar-solidified medium. (A) The number of lateral roots, (B) fresh weight of per plant, (C) chlorophyll content, and (D) primary root length of *A. thaliana*. (E-H) *A. thaliana* seedlings cocultured with strain Ac-14 in NBRIP agar-solidified medium containing 0.5 g/L $CaCO_3$. (E) The number of lateral roots, (F) fresh weight of per plant, (G) chlorophyll content, and (H) primary root length of *A. thaliana*. Different lowercase letters indicated statistically significant differences (P<0.05). *, P < 0.05; **, P < 0.01; n.s., no significant difference.

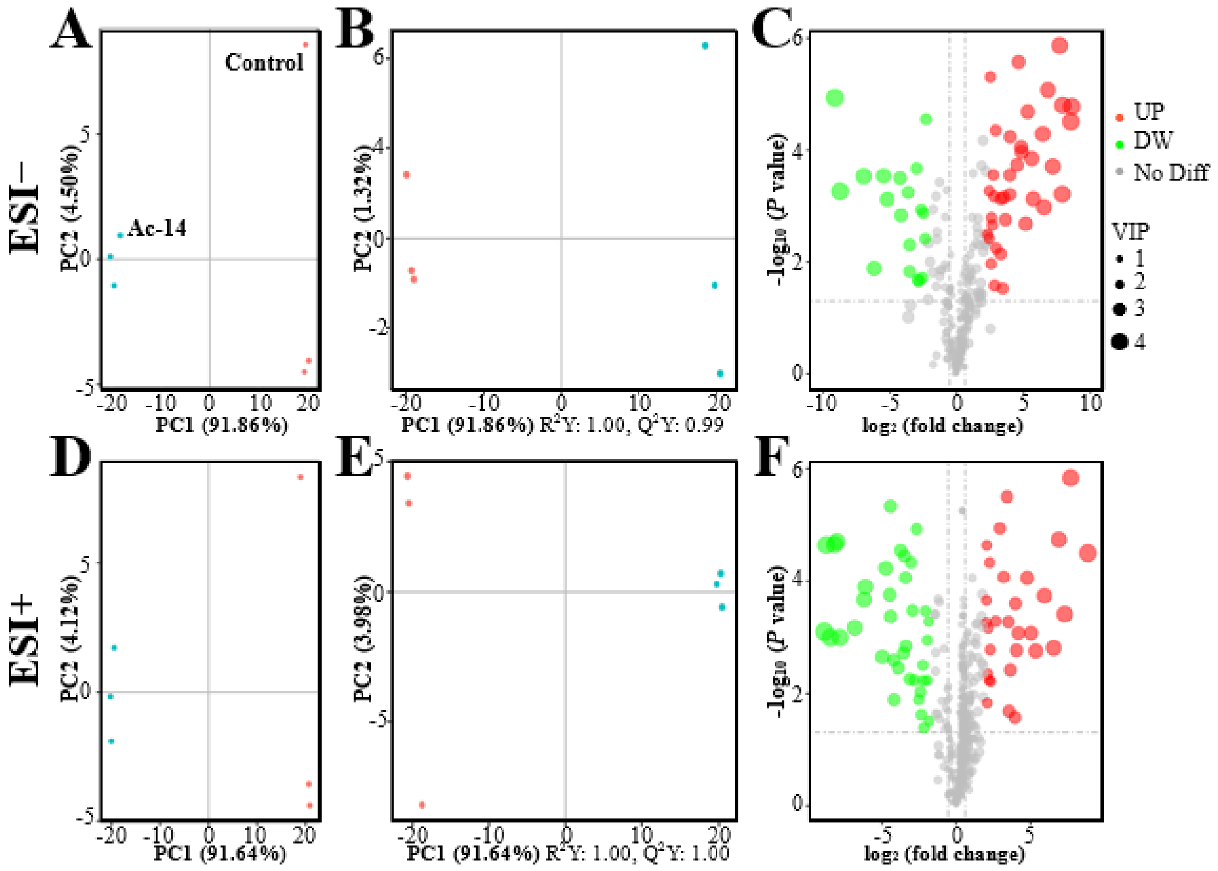

Figure S5 | Multivariate statistical analysis of untargeted metabolomics data obtained using the LC-MS/MS approach. PCA score plots of metabolomics data for *Acinetobacter* sp. Ac-14 (blue) and control (red) obtained by (A) LC-MS (ESI−), and (D) LC-MS (ESI+). PLS-DA score plots of metabolomics data obtained by (B) LC-MS (ESI−), and (E) LC-MS (ESI+). Volcano plots of (C) LC-MS (ESI−), and (F) LC-MS (ESI+). Red or green circles indicate the numbers and tendency (red, up; green, down; grey, no different) of metabolites to separate in the model when strain Ac-14 are compared with control group.

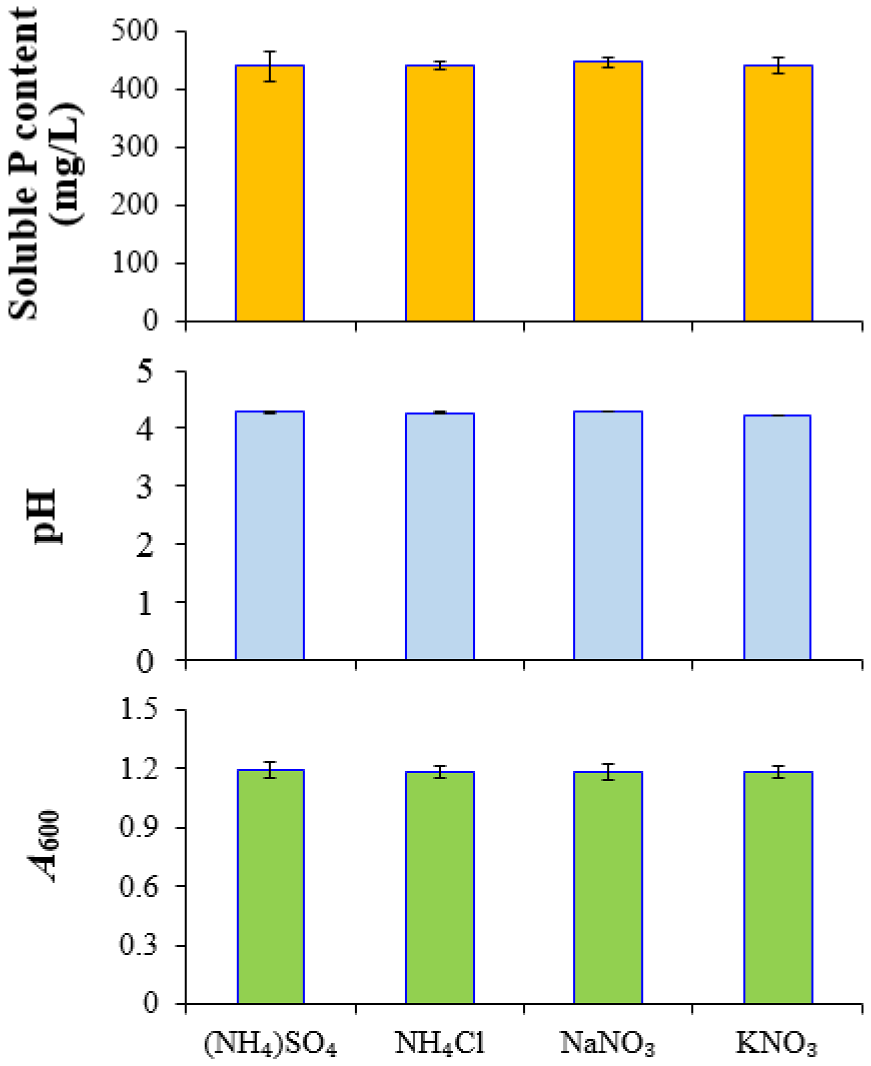

Figure S6 | The phosphate-solubilizing ability, pH value and A_{600} value of *Acinetobacter* sp. Ac-14 under different nitrogen sources.

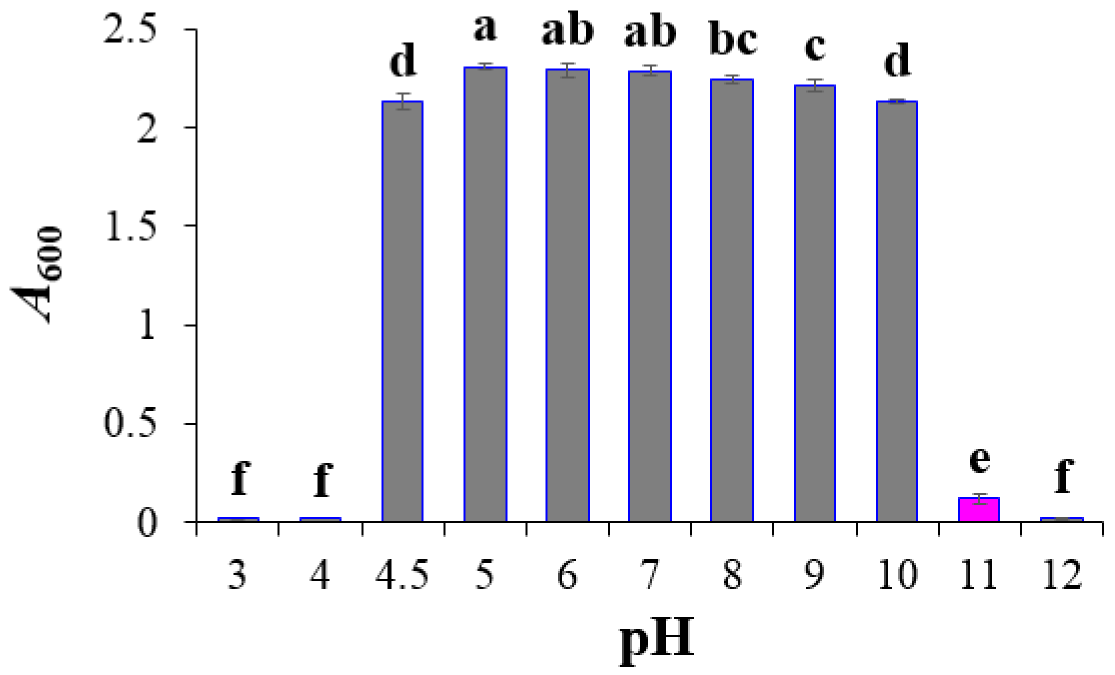

Figure S7 | Effect of different pH on the growth of *Acinetobacter* sp. Ac-14. Different lowercase letters indicated statistically significant differences ($P < 0.05$).

Supplementary “Excel S1” file

Excel S1. Detailed information about the differential metabolites and all metabolites detected by LC-MS (ESI−) and LC-MS (ESI+). (See separate “**Excel S1**”**file**)
